# The effect of probiotics on weight management in patients with severe obesity undergoing metabolic and bariatric surgery: a systematic review and meta-analysis

**DOI:** 10.1080/07853890.2025.2551284

**Published:** 2025-08-24

**Authors:** Shi Wang, Weibing Wu, Zhengwei Chen, Chaobo Xu, Kai Zhang, Xiaoya Xu

**Affiliations:** a Department of General Surgery, Lishui People’s Hospital, Lishui, China; b Department of Critical Care Medicine, Zhejiang Qingyuan People’s Hospital, Qingyuan, China; c Department of Critical Care Medicine, Second Affiliated Hospital, Zhejiang University School of Medicine, Hangzhou, China

**Keywords:** Probiotics, weight management, severe obesity, metabolic bariatric surgery, meta-analysis

## Abstract

**Background:**

Although metabolic and bariatric surgery (MBS) remains an effective intervention for severe obesity, postoperative weight regain persists as a significant clinical challenge. Probiotics have emerged as a potential adjunct therapy to optimize outcomes, but their efficacy in weight management remains controversial. This meta-analysis evaluates the effect of probiotics supplementation on weight management in patients following MBS.

**Methods:**

A comprehensive search strategy was executed across four databases (PubMed, Embase, Scopus and Cochrane Library) from inception to April 10th, 2025. Inclusion criteria encompassed randomized controlled trials comparing probiotics with placebo in patients with severe obesity undergoing MBS. Primary outcomes including percent excess weight loss (%EWL), postoperative body mass index (BMI), and BMI reduction. Data were pooled using the random-effects model.

**Results:**

A total of 13 trials included 693 patients were finally analyzed in the meta-analysis. Pooled analysis demonstrated no significant difference in %EWL (MD 0.39, 95% CI −1.90 to 2.68, I^2^=43%), postoperative BMI (MD 0.07, 95% CI −0.21 to 0.35, I^2^=26%), and BMI reduction (MD −0.05, 95% CI −0.53 to 0.44, I^2^=60%) between probiotics and control groups. Subgroup analyses stratified by surgery type, probiotic formulation and treatment duration similarly revealed no clinically meaningful effects.

**Conclusions:**

Current evidence does not support the routine use of probiotics for enhancing weight loss after MBS, regardless of surgical technique and treatment duration. Further large-scale trials standardizing strains, dosages, and outcome metrics are warranted.

## Background

Obesity poses a significant impediment to global health progress as a pervasive public health challenge. According to the recent Global Burden of Disease study, an estimated 2.11 billion adults were affected by obesity in 2021 [1]. Consequently, the burden of major non-communicable diseases (NCDs) is projected to surge. Evidence indicates that cardiovascular event incidence will more than double in some countries within the next decade [2], while diabetes cases are projected to affect over 1.31 billion individuals globally by 2050 [3]. Concurrently, obesity-related cancer diagnoses are expected to exceed 2 million new cases annually worldwide by 2070 [4]. Implementing effective interventions against obesity is therefore imperative to mitigate this escalating NCD burden. While metabolic and bariatric surgery (MBS) remains the most effective long-term intervention for severe obesity, postoperative weight regain persists as a significant clinical challenge, affecting 20 to 30% of patients [5,6], necessitating adjunct therapies to optimize outcomes [7]. The gut microbiome, profoundly altered by surgical procedures such as sleeve gastrectomy (SG) and Roux-en-Y gastric bypass (RYGB), has emerged as a critical mediator of metabolic homeostasis and weight regulation [8]. Probiotics, live microorganisms with purported benefits for gut microbial balance, have thus garnered interest as a potential strategy to amplify or sustain the metabolic advantages of MBS [9].

The gut microbiota plays a crucial role in the development of obesity by influencing energy harvest, fat storage, and systemic inflammation [10]. Studies have shown that obese individuals often have decreased microbial diversity and a higher Firmicutes-to-Bacteroidetes ratio compared to lean individuals [11]. These differences can lead to increased caloric extraction from food and metabolic dysregulation. These mechanisms highlight the rationale for using probiotics to modulate the gut microbiota as an adjunctive approach for weight management after MBS. Despite compelling preclinical data demonstrating that probiotics mitigate obesity-related dysbiosis and inflammation in animal models [12,13], clinical trials in post-bariatric populations have yielded inconsistent results [14–16]. The clinical ambiguity surrounding probiotics may stem from mechanistic complexities unique to post-surgical physiology. Bariatric procedures fundamentally alter gut anatomy, nutrient transit, and enteroendocrine signaling—factors that may modulate probiotic viability, colonization, and metabolic activity [17]. For example, a prospective observational study used the next-generation sequencing approach to compare the impact of SG and RYGB on gut bacterial microbiome and in systemic immuno-inflammatory response. The results of this study demonstrated that RYGB patients had a higher representation of family *Enterobacteriaceae* and genera *Veillonella*, while increased expression of immune-inflammatory genes was observed mainly for SG patients [18]. Furthermore, strain-specific effects are poorly characterized; *Bifidobacterium* species may regulate leptin levels to suppress appetite and alleviate postoperative gastrointestinal symptoms [19,20], whereas *Lactobacillus* strains could exert anti-inflammatory effects in the surgically reshaped stomach [21].

A recent meta-analysis by Rakab et al. evaluated probiotic/synbiotic supplementation after bariatric surgery but primarily focused on cardiometabolic outcomes [22]. Therefore, this meta-analysis aims to synthesize evidence from randomized controlled trials (RCTs) to evaluate the effect of probiotics supplementation in weight management following MBS. Specifically, we address the critical knowledge gap that how strain specificity, surgery type and intervention duration influence outcomes. By delineating these factors, our findings seek to inform evidence-based recommendations for probiotic use in post-bariatric care and guide future research toward personalized microbiota-targeted therapies.

## Methods

### Search strategy and study selection

This study adhered to the 2020 PRISMA (Preferred Reporting Items for Systematic Reviews and Meta-Analyses) guidelines [23], with the completed checklist provided in Supplementary Material 1. The protocol was prospectively registered with the Open Science Framework (https://osf.io/k8gz9). Two investigators independently executed a systematic search across PubMed, Embase, Scopus, and Cochrane CENTRAL until April 10th, 2025. The search included keywords such as ‘probiotics’, ‘bariatric’, ‘gastric bypass’, ‘sleeve gastrectomy’, and ‘randomized’. The detailed search strategies are given in Supplementary Material 2.

Eligibility criteria encompassed:

1. Population: Adults (≥18 years) with severe obesity (BMI ≥ 40 kg/m^2^ or 35 ≤ BMI ≤ 40 kg/m^2^ with pertinent obesity-related diseases) undergoing MBS including RYGB, Mini Gastric Bypass, One-Anastomosis Gastric Bypass, and SG.
2. Intervention: Probiotic supplementation (any strain, dose, or duration) initiated perioperatively.
3. Comparator: Placebo or standard care without probiotics.
4. Outcomes: Primary outcomes included percent excess weight loss (%EWL), postoperative body mass index (BMI), and BMI reduction.
5. Study Design: RCTs published in English.

Exclusion criteria included: duplicate publications, studies involving non-bariatric surgical cohorts or non-obese populations; case reports, non-human studies, non-randomized studies, studies failing to report quantifiable weight loss metrics in extractable format.

### Data extraction

Two investigators (Shi Wang and Weibing Wu) independently conducted the literature search and screening process. After deduplication, titles and abstracts were screened against predefined eligibility criteria. Potentially relevant studies underwent full-text review, with discrepancies resolved through iterative discussion until consensus was achieved. A third author (Zhengwei Chen) adjudicated unresolved conflicts. Two investigators independently extracted data using a standardized form, including the first author, publication year, sample size, population characteristics, probiotic regimen (strain, dosage, duration), outcome measures and timepoints. If data were unavailable in the trial report or data collection, we contacted the corresponding authors to obtain essential missing information.

### Quality assessment

Methodological quality was independently assessed by two reviewers (Shi Wang and Weibing Wu) using the Cochrane risk of bias tool [24]. Publication bias was assessed using the Egger’s regression test and visual inspection of funnel plots [25]. When asymmetry suggested small-study effects, the trim-and-fill method was employed to further assess its potential impact on our meta-analysis [26]. Any discrepancies throughout all phases were ultimately resolved through team consensus.

### Statistical synthesis and analysis

Quantitative synthesis was performed using Review Manager Version 5.3 and R software (‘meta’ package). Continuous outcomes were analyzed as mean differences (MDs) with 95% confidence intervals (CIs), calculated *via* the inverse-variance weighting method. A random-effects model was applied to account for clinical and methodological heterogeneity. Heterogeneity was quantified using I^2^ statistics, with values > 50% indicating substantial heterogeneity [27]. Predefined subgroup analyses were conducted to explore sources of heterogeneity: surgery type (SG versus gastric bypass), probiotic formulation (single-strain versus multi-strain), treatment duration (< 3 months versus ≥ 3 months).

Additionally, we conducted a sensitivity analysis to assess the impact of individual studies by omitting one at a time. A p-value < 0.05 was considered statistically significant.

## Results

### Study selection and study characteristics

**Figure 1.**
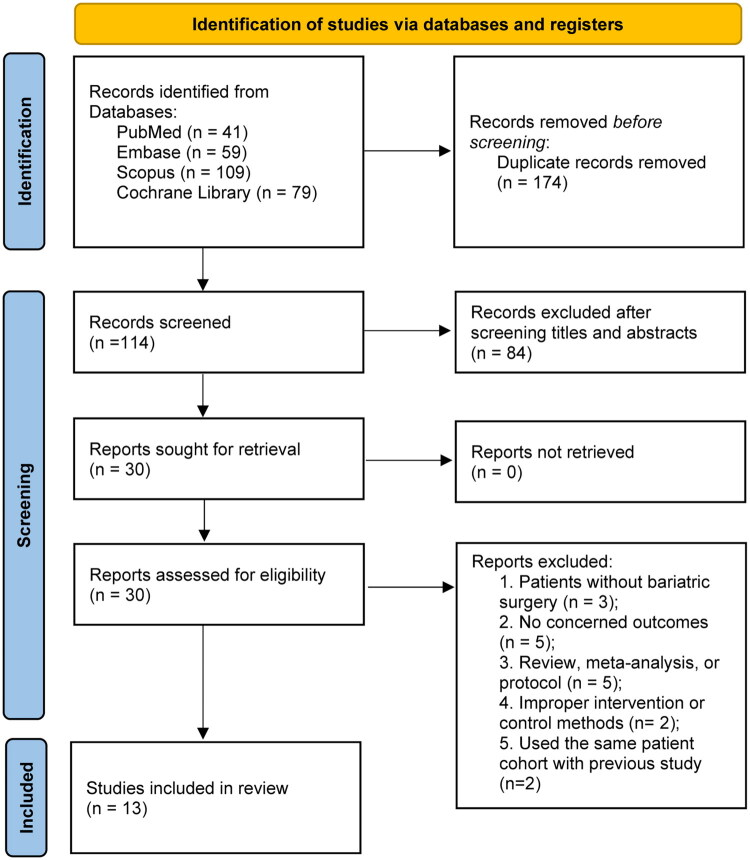
PRISMA 2020 flow diagram for this meta-analysis.

The study identification and screening process is detailed in the PRISMA-mandated flowchart (Figure 1). Our systematic search initially retrieved 288 records from four databases: PubMed (*n* = 41), Embase (*n* = 59), Scopus (*n* = 109), and Cochrane Library (*n* = 79). Initially, all records were imported into a document management software, and 174 duplicated articles were electronically removed. Subsequent title and abstract screening excluded 84 studies; full-text evaluation further excluded 17 studies for various reasons. Finally, our meta-analysis included a total of 13 studies [28–39], encompassing 693 patients with severe obesity following MBS (337 in the probiotic group and 356 in the placebo group).

Table 1 summarizes the basic characteristics of the included studies. The studies were published between 2009 and 2024. Patients with severe obesity who undergoing MBS were included in this analysis, including RYGB in 10 studies [28,30,31,33–36,38–40], and SG in 3 studies[29,32,37]. In two studies [35,36], baseline BMI was between 35 and 40 kg/m^2^, other studies enrolled patients with baseline BMI greater than 40 kg/m^2^. The sample size in each study varies from 6 to 135 patients. The treatment duration within each of these studies varied as well. In one study intervention was applied from 1 month preoperatively to 3 months postoperatively[34], in another study intervention was applied for 3 months preoperatively [32]. The remaining 11 studies applied intervention postoperatively, the treatment duration was 15 days in one study[36], 1 month in one study [37], 12 weeks in studies [31,35,38], 3 months in studies [28,30,33,39], and 6 months in two studies [29,40]. In one of the studies [40], a single probiotic (Lactobacillus) was used, whereas other studies used multiple probiotics.

**Table 1.** Characteristics of included studies.

Study	Sample size	Population	Intervention and control methods	Outcomes
Potrykus 2024 [32]	22/26	Adult patients were qualified for laparoscopic sleeve gastrectomy or one anastomosis gastric bypass (baseline BMI: 40.5 kg/m^2^)	Intervention: Bifidobacterium bifidum W23, Bifidobacterium lactis W51 and W52, Lactobacillus acidophilus W37, Levilactobacillus brevis W63, Lacticaseibacillus casei W56, Ligilactobacillus salivarius W24, Lactococcus lactis W19, and Lactococcus lactis W58 in daily dose of 2 × 10^9^ CFU, started at 12 weeks before surgery, for 12 weeks; Control: placebo tablets (maize starch and maltodextrin of maize origin)	BMI, BMI reduction, %EWL at 6 months
Melali 2024 [33]	68/67	Adult patients with severe obesity, were candidates for Roux-en-Y gastric bypass (baseline BMI: 46.3 kg/m^2^)	Intervention: probiotic supplements (Familact), started at 1 week after surgery, for 3 months; Control: placebo	BMI, BMI reduction, %EWL at 6 months
Ghafouri-Taleghani 2024 [35]	20/21	Adult patients with food addiction and weight regain after metabolic bariatric surgery (baseline BMI: 35 kg/m^2^)	Intervention: Lactobacil lus acidophilus (1.8 × 10^9^ CFU/capsule), Bifidobacterium bifidum (1.8 × 10^9^ CFU/capsule), Bifidobacterium lactis (1.8 × 10^9^ CFU/capsule), Bifdobacterium longum (1.8 × 10^9^ CFU/capsule), Lactobacillus reuteri (1 × 10^9^ CFU/capsule), Lactobacillus rhamnosus (1 × 10^9^ CFU/capsule), started at 1 week after surgery, for 12 weeks; Control: placebo tablets (300 mg of starch)	BMI, BMI reduction at 12 weeks
Dowgiałło-Gornowicz 2024 [37]	15/16	Adult patients qualified for sleeve gastrectomy and without specific symptoms of gastrointestinal tract diseases (baseline BMI: 41.5 kg/m^2^)	Intervention: Lactobacillus plantarum (5 × 10^8^ cfu/g), Bifidobacterium animalis (1 × 10^10^ cfu/g) and Bifidobacterium breve (1 × 10^10^ cfu/g), started at 1 week after surgery, for 1 month; Control: placebo tablets	BMI, BMI reduction, %EWL at 1 month
Ramos 2022 [30]	13/16	Adult patients with BMI ≥35 kg/m^2^, were candidates for Roux-Y gastric bypass (baseline BMI: 43 kg/m^2^)	Intervention: 5 billion Lactobacillus acidophilus NCFM Strain and 5 billion Bifidobacterium lactis Bi-07 per day, started at 7-day after surgery, for 90 days; Control: placebo tablets (starch and lactose)	BMI, BMI reduction, %EWL at 90 days
Crommen 2022 [38]	25/23	Adult patients (≥20 years old) with BMI ≥35 kg/m^2^, were scheduled to undergo gastric bypass surgery (baseline BMI: 43.2 kg/m^2^)	Intervention: Lactobacillus acidophilus, Bifidobacterium breve, B. longum, L. delbrueckii susp. bulgaricus, L.helveticus, L. plantarum, L. rhamnosus, L. casei, Lactococcus lactis susp. lactis, and Streptococcus thermophiles of 15 × 10^9^ CFU/4 g per day, started at 2-day after discharge from hospital, for 12 weeks; Control: placebo tablets (corn dextrin and rice starch)	BMI, BMI reduction at 12 weeks
Carlos 2022 [39]	22/22	Adult patients with BMI ≥35 kg/m^2^, were scheduled to undergo Roux-en-Y gastric bypass (baseline BMI: 43.1 kg/m^2^)	Intervention: 5 billion Lactobacillus acidophilus and 5 billion Bifidobacterium lactis, started after surgery, for 90 days; Control: placebo tablets	BMI, BMI reduction at 1 year
Wagner 2021 [28]	34/39	Adult patients with BMI ≥35 kg/m^2^, undergoing Roux-en-Y gastric bypass (baseline BMI: 41.7 kg/m^2^)	Intervention: 5 billion Lactobacillus acidophilus and 5 billion Bifidobacterium lactis, started after surgery, for 90 days; Control: placebo tablets	%EWL at 3 months
Ramos 2021 [31]	33/38	Adult patients with BMI ≥35 kg/m^2^, undergoing Roux-en-Y gastric bypass (baseline BMI: 44.1 kg/m^2^)	Intervention: 5 billion Lactobacillus acidophilus and 5 billion Bifidobacterium lactis per day, started at 7 days after surgery, for 12 weeks; Control: placebo tablets (starch and lactose)	BMI, BMI reduction, %EWL at 12 weeks
Karbaschian 2018 [34]	23/23	Adult morbidly patients with obesity undergoing for the laparoscopic one anastomosis gastric bypass-mini gastric bypass surgery (baseline BMI: 44.7 kg/m^2^)	Intervention: Lactobacillus casei (3.5 × 10^9^ CFU/g), Lactobacillus rhamnosus (7.5 × 10^8^ CFU/g), Streptococcus thermophilus (1 × 10^8^ CFU/g), Bifidobacterium breve (1 × 10^10^ CFU/g), Lactobacillus acidophilus (1 × 10^9^ CFU/g), Bifidobacterium longum (3.5 × 10^9^ CFU/g), and Lactobacillus bulgaricus (1 × 10^8^ CFU/g), started 4 weeks before surgery to 12 weeks after surgery; Control: placebo tablets (maltodextrin)	BMI, BMI reduction, %EWL at 16 weeks
Sherf-Dagan 2017 [29]	40/40	Adult morbidly patients with obesity undergoing laparoscopic sleeve gastrectomy (baseline BMI: 42.1 kg/m^2^)	Intervention: 25 billion live cells of Lactobacillus acidophilus, Bifidobacterium bifidum, Lactobacillus rhamnosus, Lactococcus lactis, Lactobacillus casei, Bifidobacterium breve, Streptococcus thermophiles, Bifidobacterium longum, Lactobacillus paracasei, Lactobacillus plantarum, Bifidobacterium infatis, started after surgery, for 6 months; Control: placebo tablets	BMI, %EWL at 1 year
Fernandes 2016 [36]	3/3	Adult patients with severe obesity undergoing Roux-en-Y gastric bypass (baseline BMI: 39.4 kg/m^2^)	Intervention: 1 × 10^9^ Lactobacillus paracasei, 1 × 10^9^ Lactobacillus rhamnosus, 1 × 10^9^ Lactobacillus acidophilus, and 1 × 10^9^ Bifidobacteri um lactis, started after surgery, for 15 days; Control: placebo tablets (maltodextrin)	BMI, BMI reduction, %EWL at 15 days
Woodard 2009 [40]	19/22	Adult morbidly patients with obesity undergoing Roux-en-Y gastric bypass (baseline BMI: 47.6 kg/m^2^)	Intervention: 2.4 billion live cells of Lactobacillus species, started after surgery, for 6 months; Control: placebo tablets	%EWL at 6 months

### Quality assessment

**Figure 2.**
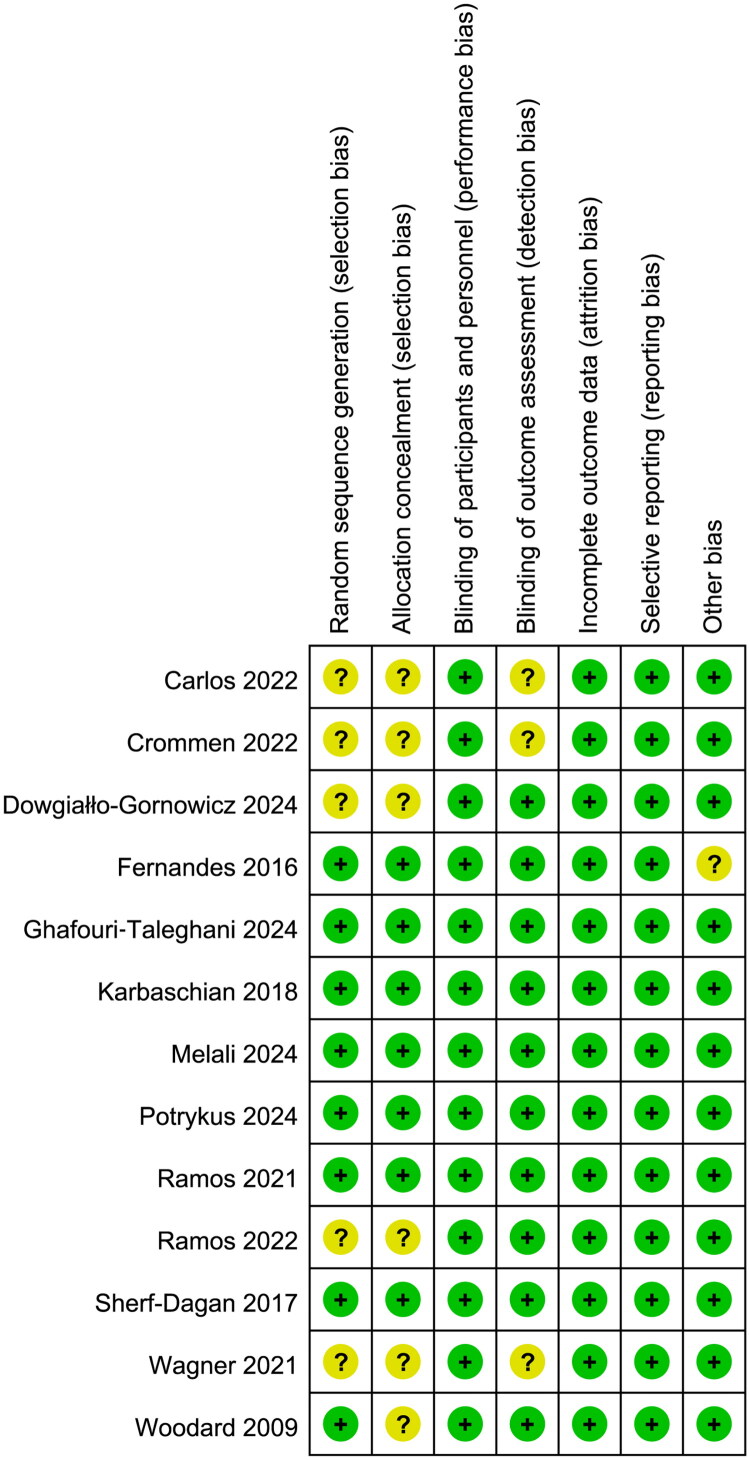
Assessment of quality by the cochrane risk of bias tool. Red denotes high risk, yellow unclear risk and green low risk.

Figure 2 provides a depiction of the quality assessment conducted utilizing the Cochrane risk of bias tool. Notably, all trials were double-blind trials, they had low risk of performance bias. Additionally, six studies [28,30,37–40] failed to furnish details regarding random sequence generation or allocation concealment. Regarding the blinding method for outcome assessment, three studies [28,38,39] demonstrated unclear risk due to incomplete descriptions of outcome assessor blinding, potentially inflating effect estimates. Moreover, in one study [36], the treatment duration and follow-up period were much shorter than other studies, which could have contributed to other bias.

Visual inspection of the funnel plot (Supplementary Material 3) revealed symmetrical distribution of effect sizes around the pooled estimate. Statistical confirmation *via* Egger’s linear regression test indicated no significant small-study effects.

### Meta‐analysis results

**Figure 3.**
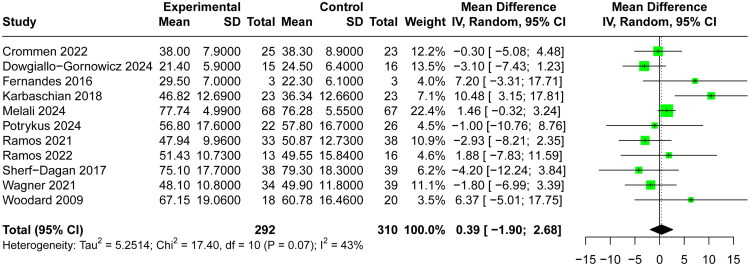
Forest Plot comparing the effect of probiotic versus control groups for %EWL.

**Figure 4.**
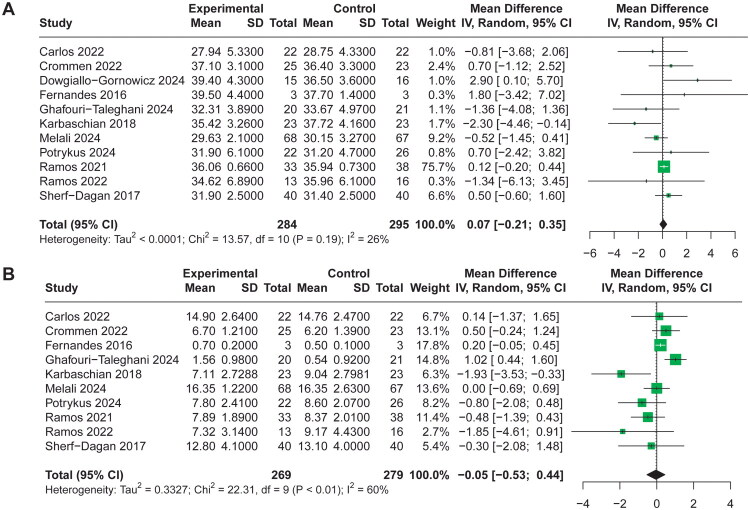
Forest Plot comparing the effect of probiotic versus control groups for (A) postoperative BMI, (B) BMI reduction.

A total of 11 included RCTs [28–34,36–38,40] reported %EWL at the end of follow‑up, pooled analysis using a random‑effects model demonstrated no significant difference between probiotic and control groups for %EWL (MD 0.39, 95% CI −1.90 to 2.68, I^2^=43%, Figure 3). Furthermore, 11 RCTs [29–39] reported postoperative BMI and 10 RCTs [29–36,38,39] reported BMI reduction at the end of follow‑up, respectively. The random-effects model result indicated that the use of probiotics had no significant effect on postoperative BMI (MD 0.07, 95% CI −0.21 to 0.35, I^2^=26%, Figure 4A) and BMI reduction (MD −0.05, 95% CI −0.53 to 0.44, I^2^=60%, Figure 4B). Sensitivity analyses excluding each study in turn did not materially alter the overall estimate (Supplementary Material 4), indicating stability of the result.

**Figure 5.**
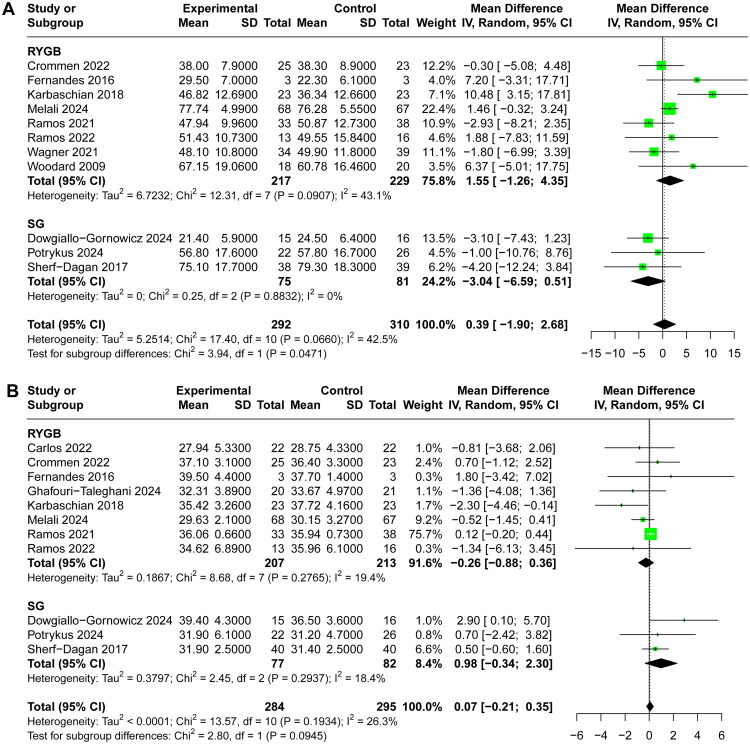
Subgroup analysis stratified by surgery type for (A) %EWL, (B) postoperative BMI.

In patients undergoing gastric bypass, probiotics supplementation was associated with a non-significant trend toward greater %EWL compared to placebo (MD 1.55, 95% CI −1.26 to 4.35, I^2^=43%, Figure 5A). Conversely, SG recipients receiving probiotics showed lower %EWL (MD −3.04, 95% CI −6.59 to 0.51, I^2^=0%, Figure 5A) and greater postoperative BMI (MD 0.98, 95% CI −0.34 to 2.30, I^2^=18%, Figure 5B) compared to placebo, though this difference similarly failed to reach statistical significance.

Moreover, subgroup analyses stratified by probiotic formulation (single-strain versus multi-strain), treatment duration (< 3 months versus ≥ 3 months) yielded no significant associations between probiotics supplementation and improved %EWL, postoperative BMI, or BMI reduction (Supplementary Material 5-7).

## Discussion

This meta-analysis evaluating the effect of probiotics in weight management among post-MBS patients revealed no statistically significant benefits of probiotics supplementation on key outcomes, including %EWL, postoperative BMI, or BMI reduction at the end of follow‑up. These results suggest that the routine use of probiotics solely for the purpose of augmenting weight loss after bariatric procedures may not be justified based on current evidence.

To the best of our knowledge, this study represents the most up-to-date meta-analysis evaluating the effects of probiotic supplementation on weight management in patients with severe obesity undergoing MBS. A total of 13 studies, comprising 693 patients with severe obesity who underwent MBS, were included in the final analysis. The results indicated that probiotic supplementation did not confer significant benefits in terms of either %EWL or BMI reduction. Collectively, our findings are consistent with and reinforce those reported in previous meta-analyses [16,41], thereby strengthening the current body of evidence. Swierz et al. [16] analyzed five RCTs, indicated that probiotic supplementation have no significant effect regarding %EWL and quality of life, but might alleviate some gastrointestinal symptoms. Subsequently, Zhang and colleagues [41] demonstrated that probiotic supplementation help patients with severe obesity in achieving further waist circumference improvement after MBS, but with no significant effect on weight, BMI, and %EWL. Moreover, Wang et al. [14] and Chen et al. [42] reported that probiotic supplementation in patients with severe obesity undergoing MBS exerted beneficial effects on several metabolic and nutritional indicators, including regulation of aspartate aminotransferase, triglyceride levels, food intake, and vitamin B12 status. Recently, Rakab et al. [22] performed an updated systematic review and meta-analysis of 13 RCTs, indicating that probiotics/synbiotics supplementation could improve cardiometabolic outcomes including glycemic control, lipid profile, liver enzymes, and vitamin D levels. Additionally, Suzumura et al. [15] assessed the effects of probiotics or synbiotics among overweight and adults with obesity who did not undergo surgery, suggested that probiotics or synbiotics supplementation may slightly reduce waist circumference, but has no significant effect on body weight or BMI.

A possible explanation for the lack of significant benefit observed in our analysis is the heterogeneity in probiotic strains, dosages, and treatment durations among the included studies. Probiotics are a diverse group of microorganisms with strain-specific effects. The absence of standardization across trials complicates the interpretation and generalization of findings. Some studies included in this meta-analysis administered single-strain formulations, while others used multi-strain combinations. The duration of intervention varied from a few weeks to several months, which may have been insufficient to induce a sustained metabolic effect. Additionally, many studies lacked baseline or follow-up microbiota profiling, making it difficult to determine whether probiotics effectively colonized the gut or led to meaningful microbiome changes[43].

Notably, our subgroup analyses demonstrated that probiotic supplementation was associated with greater %EWL in patients undergoing RYGB compared to placebo, whereas reduction in %EWL was observed in patients undergoing SG. RYGB induces profound anatomical and functional alterations in the gastrointestinal tract, including duodenal exclusion, accelerated distal gut nutrient delivery, and subsequent microbial remodeling[44,45]. These changes may create a microenvironment conducive to probiotic colonization and metabolic modulation. Probiotics may enhance weight loss post-RYGB through mechanisms such as bile acid deconjugation, short-chain fatty acid production, and amelioration of surgery-induced dysbiosis, thereby potentiating metabolic benefits[46,47]. Furthermore, reduced gastric acidity post-RYGB might improve probiotic viability[48], facilitating sustained microbial engraftment. Conversely, SG primarily restricts gastric volume without significant anatomical rearrangement of the intestinal tract[49]. The maintenance of normal gastro-intestinal continuity and relatively intact enteric neurohormonal axes in patients undergoing SG might limit the opportunity for exogenous probiotics to exert clinically meaningful metabolic effects [49,50]. The observed attenuation of %EWL in SG cohorts could reflect competitive interactions between supplemented probiotics and the less disrupted native microbiota, potentially diminishing beneficial symbiont activity. Additionally, differential postoperative alterations in ghrelin secretion or glucose homeostasis between procedures may modulate probiotic efficacy. Emerging microbiome research supports this interpretation. A recent review by Voermans et al. highlighted that MBS (especially RYGB) induces substantial shifts in gut microbiota, including increased microbial diversity and enrichment of taxa such as *Akkermansia muciniphila*, *Veillonella*, and *Streptococcus*, which are associated with improved metabolic parameters [51]. These microbial changes may contribute to the metabolic benefits of surgery and enhance the host environment for probiotic engraftment. In contrast, the more modest microbiota alterations seen after SG may partly explain the limited efficacy of probiotics observed in SG patients.

In addition, probiotics may offer other potential benefits to post-MBS patients, such as improved gastrointestinal symptoms, enhanced micronutrient absorption, modulation of systemic inflammation, and restoration of microbial diversity [14]. Some included studies reported reduced small intestinal bacterial overgrowth (SIBO), lower risk of gall bladder disease, improved vitamin B12 levels and postoperative gastrointestinal symptoms with probiotic use [32,52,53]. However, a recent meta-analysis analyzed five RCTs showed that probiotics did not offer significant benefits in treating SIBO in patients after MBS [54]. This negative result may reflect the limited number of available trials rather than a true lack of efficacy. The current evidence base remains insufficient to draw definitive conclusions, and larger, well-designed RCTs are needed to adequately assess the therapeutic potential of probiotics for post-MBS SIBO prevention and treatment.

Furthermore, postoperative gastrointestinal symptoms such as constipation and bloating are common for patients undergoing MBS [55]. A study indicated that probiotics may help reduce the risk of postoperative constipation, which is a common concern after bariatric surgery due to dietary changes and altered gut motility [37]. The anti-constipation effects of probiotics operate through multiple physiological pathways that are particularly relevant in the post-MBS population. They increase intestinal water retention and stool bulk by producing organic acids that lower colonic pH and support the growth of beneficial bacteria [56]. Certain strains also modulate the enteric nervous system by producing neurotransmitters like GABA, which enhance gut motility. Moreover, probiotics help restore microbial diversity often disrupted by surgery and dietary changes, supporting fiber fermentation, mucus production, and intestinal barrier integrity [57].

### Future research directions

Future research should prioritize strain-specific RCTs incorporating multi-omics approaches to identify patient subgroups with dysbiosis patterns amenable to probiotic modulation [58]. Well-designed, adequately powered RCTs should focus on strain-specific effects and standardized dosing regimens, ideally supported by multi-omics approaches such as metagenomics, metabolomics, and transcriptomics to clarify host-microbiome interactions after bariatric surgery. Investigating synbiotics formulations or genetically engineered probiotics targeting post-bariatric metabolic pathways (e.g. farnesoid X receptor and G-protein-coupled bile acid receptor signaling) may yield more clinically relevant effects [59]. Additionally, extended follow-up periods are critical to assess whether microbiome stabilization impacts long-term weight regain, a prevalent challenge in bariatric care. Besides, longer follow-up periods are also essential to evaluate whether sustained probiotic supplementation can reduce weight regain, improve metabolic parameters, and prevent gastrointestinal complications such as SIBO or constipation.

### Strength and limitations

The strengths of this meta-analysis include a rigorous and systematic search strategy, inclusion of only randomized controlled trials, and separate evaluation of both %EWL and BMI as distinct outcome measures. Nonetheless, several limitations must be acknowledged. First, the number of eligible studies was relatively small, and most trials had small sample sizes with short-term follow-up (<12 months), which limits the statistical power to detect small but potentially relevant effects. Second, variability in adjunct interventions (e.g. dietary protocols, physical activity counseling) across trials may have introduced confounding. Third, the lack of standardized fecal microbiome profiling in most studies precludes evaluation of whether probiotic-induced microbial shifts correlate with clinical outcomes. To obtain more robust evidence, further high-quality, prospective, multicenter randomized controlled studies with larger sample sizes are warranted.

## Conclusion

In conclusion, the current evidence does not support a significant effect of probiotic supplementation on %EWL or BMI in patients undergoing MBS. While probiotics may have other potential clinical benefits, their use should not be promoted solely for enhancing postoperative weight loss based on existing data. Well-designed, large-scale RCTs with standardized probiotic formulations and longer follow-up durations are needed to elucidate the role of probiotics in the complex physiological milieu following MBS.

## Supplementary Material

sup5subewl.tif

sfile2 search.docx

sup4 sen.tif

sfile1 PRISMA.docx

sup6subbmi.tif

sup7subbmi2.tif

sup3 funnel.tif

## Data Availability

All data generated or analyzed during this study are included in this published article and its supplementary information files. Further data can be requested from the corresponding author.

## References

[1] Global, regional, and national prevalence of adult overweight and obesity, 1990-2021, with forecasts to 2050: a forecasting study for the Global Burden of Disease Study 2021. Lancet. 2025;405(10481):813–838. 40049186 10.1016/S0140-6736(25)00355-1 PMC11920007

[2] Mohebi R, Chen C, Ibrahim NE, et al. Cardiovascular disease projections in the united states based on the 2020 census estimates. J Am Coll Cardiol. 2022;80(6):565–578. doi: 10.1016/j.jacc.2022.05.033. 35926929 PMC9396356

[3] Global, regional, and national burden of diabetes from 1990 to 2021, with projections of prevalence to 2050: a systematic analysis for the Global Burden of Disease Study 2021. Lancet. 2023;402(10397):203–234. 37356446 10.1016/S0140-6736(23)01301-6 PMC10364581

[4] Soerjomataram I, Bray F. Planning for tomorrow: global cancer incidence and the role of prevention 2020-2070. Nat Rev Clin Oncol. 2021;18(10):663–672. doi: 10.1038/s41571-021-00514-z. 34079102

[5] Courcoulas AP, Daigle CR, Arterburn DE. Long term outcomes of metabolic/bariatric surgery in adults. BMJ. 2023;383:e071027. doi: 10.1136/bmj-2022-071027. 38110235

[6] Noria SF, Shelby RD, Atkins KD, et al. Weight regain after bariatric surgery: scope of the problem, causes, prevention, and treatment. Curr Diab Rep. 2023;23(3):31–42. doi: 10.1007/s11892-023-01498-z. 36752995 PMC9906605

[7] Cohen RV, Petry TB. How to address weight regain after bariatric surgery in an individualized way. Rev Endocr Metab Disord. 2023;24(5):993–1002. doi: 10.1007/s11154-023-09806-4. 37171756

[8] Gasmi A, Bjørklund G, Mujawdiya PK, et al. Gut microbiota in bariatric surgery. Crit Rev Food Sci Nutr. 2023;63(28):9299–9314. doi: 10.1080/10408398.2022.2067116. 35531940

[9] Santos-Paulo S, Costello SP, Forster SC, et al. The gut microbiota as a therapeutic target for obesity: a scoping review. Nutr Res Rev. 2022;35(2):207–220. doi: 10.1017/S0954422421000160. 34100344

[10] Baek KR, Singh S, Hwang HS, et al. Using gut microbiota modulation as a precision strategy against obesity. Int J Mol Sci. 2025;26(13):6282. doi: 10.3390/ijms26136282. 40650060 PMC12249903

[11] Enache RM, Profir M, Roşu OA, et al. The role of gut microbiota in the onset and progression of obesity and associated comorbidities. Int J Mol Sci. 2024;25(22):12321. doi: 10.3390/ijms252212321. 39596385 PMC11595101

[12] Zhao C, Xie L, Shen J, et al. Lactobacillus acidophilus YL01 and its exopolysaccharides ameliorate obesity and insulin resistance in obese mice via modulating intestinal specific bacterial groups and AMPK/ACC signaling pathway. Int J Biol Macromol. 2025;300:140287. doi: 10.1016/j.ijbiomac.2025.140287. 39863204

[13] Lim SYM, Chong EJ, Mah WY, et al. Exploring the anti-obesity effects of Lactobacillus in C57BL/6 mice: mechanisms, interventions, and future directions. Lett Appl Microbiol. 2025;78(3) doi: 10.1093/lambio/ovaf024. 39965784

[14] Wang Y, Zheng Y, Kuang L, et al. Effects of probiotics in patients with morbid obesity undergoing bariatric surgery: a systematic review and meta-analysis. Int J Obes (Lond). 2023;47(11):1029–1042. doi: 10.1038/s41366-023-01375-5. 37674033 PMC10600003

[15] Suzumura EA, Bersch-Ferreira ÂC, Torreglosa CR, et al. Effects of oral supplementation with probiotics or synbiotics in overweight and obese adults: a systematic review and meta-analyses of randomized trials. Nutr Rev. 2019;77(6):430–450. doi: 10.1093/nutrit/nuz001. 30924853

[16] Swierz MJ, Storman D, Staskiewicz W, et al. Efficacy of probiotics in patients with morbid obesity undergoing bariatric surgery: a systematic review and meta-analysis. Surg Obes Relat Dis. 2020;16(12):2105–2116. doi: 10.1016/j.soard.2020.08.038. 33069600

[17] Gutiérrez-Repiso C, Moreno-Indias I, Tinahones FJ. Shifts in gut microbiota and their metabolites induced by bariatric surgery. Impact of factors shaping gut microbiota on bariatric surgery outcomes. Rev Endocr Metab Disord. 2021;22(4):1137–1156. doi: 10.1007/s11154-021-09676-8. 34287758

[18] Lazaro A, Tiago I, Mendes J, et al. Sleeve gastrectomy and gastric bypass impact in patient’s metabolic, gut microbiome, and immuno-inflammatory profiles-a comparative study. Obes Surg. 2025;35(3):733–745. doi: 10.1007/s11695-025-07708-9. 39870942 PMC11906558

[19] Tian P, Zou R, Wang L, et al. Multi-probiotics ameliorate major depressive disorder and accompanying gastrointestinal syndromes via serotonergic system regulation. J Adv Res. 2023;45:117–125. doi: 10.1016/j.jare.2022.05.003. 35618633 PMC10006521

[20] Lee M, Bok MK, Son K, et al. Bifidobacterium lactis IDCC 4301 (B. lactis Fit^™^) supplementation effects on body fat, serum triglyceride, and adipokine ratio in obese women: a randomized clinical trial. Food Funct. 2024;15(16):8448–8458. doi: 10.1039/d4fo00535j. 39051504

[21] Xu B, Kong J, Lin Y, et al. Anti-Helicobacter pylori activity and gastroprotective effects of human stomach-derived Lactobacillus paragasseri strain LPG-9. Food Funct. 2023;14(24):10882–10895. doi: 10.1039/d3fo03562j. 37987614

[22] Rakab MS, Rateb RM, Maamoun A, et al. Impact of probiotic/synbiotic supplementation on post-bariatric surgery anthropometric and cardiometabolic outcomes: an updated systematic review and meta-analysis of randomized controlled trials. Nutrients. 2025;17(13):2193. doi: 10.3390/nu17132193. 40647296 PMC12251824

[23] Page MJ, McKenzie JE, Bossuyt PM, et al. The PRISMA 2020 statement: an updated guideline for reporting systematic reviews. BMJ. 2021;372:n71. doi: 10.1136/bmj.n71. 33782057 PMC8005924

[24] Higgins JP, Altman DG, Gøtzsche PC, et al. The Cochrane Collaboration’s tool for assessing risk of bias in randomised trials. BMJ. 2011;343(oct18 2):d5928–d5928. doi: 10.1136/bmj.d5928. 22008217 PMC3196245

[25] Egger M, Davey Smith G, Schneider M, et al. Bias in meta-analysis detected by a simple, graphical test. BMJ. 1997;315(7109):629–634. doi: 10.1136/bmj.315.7109.629. 9310563 PMC2127453

[26] Duval S, Tweedie R. Trim and fill: a simple funnel-plot-based method of testing and adjusting for publication bias in meta-analysis. Biometrics. 2000;56(2):455–463. doi: 10.1111/j.0006-341x.2000.00455.x. 10877304

[27] Higgins JP, Thompson SG, Deeks JJ, et al. Measuring inconsistency in meta-analyses. BMJ. 2003;327(7414):557–560. doi: 10.1136/bmj.327.7414.557. 12958120 PMC192859

[28] Wagner NRF, Ramos MRZ, de Oliveira Carlos L, et al. Effects of Probiotics Supplementation on Gastrointestinal Symptoms and SIBO after Roux-en-Y Gastric Bypass: a Prospective, Randomized, Double-Blind, Placebo-Controlled Trial. Obes Surg. 2021;31(1):143–150. doi: 10.1007/s11695-020-04900-x. 32780258

[29] Sherf-Dagan S, Zelber-Sagi S, Zilberman-Schapira G, et al. Probiotics administration following sleeve gastrectomy surgery: a randomized double-blind trial. Int J Obes (Lond). 2018;42(2):147–155. doi: 10.1038/ijo.2017.210. 28852205

[30] Ramos MRZ, Felicidade I, de Oliveira Carlos L, et al. Effect of probiotic supplementation on plasma metabolite profile after Roux-Y gastric bypass: a prospective, randomized, double-blind, placebo-controlled clinical trial. Int J Obes (Lond). 2022;46(11):2006–2012. doi: 10.1038/s41366-022-01213-0. 35987956

[31] Ramos MRZ, de Oliveira Carlos L, Wagner NRF, et al. Effects of Lactobacillus acidophilus NCFM and bifidobacterium lactis Bi-07 supplementation on nutritional and metabolic parameters in the early postoperative period after roux-en-Y gastric bypass: a randomized, double-blind, placebo-controlled trial. Obes Surg. 2021;31(5):2105–2114. doi: 10.1007/s11695-021-05222-2. 33443719

[32] Potrykus M, Czaja-Stolc S, Stankiewicz M, et al. Preoperative multistrain probiotic supplementation does not affect body weight changes or cardiometabolic risk factors in bariatrics: randomized, double-blind, placebo-controlled clinical trial. Nutrients. 2024;16(13):2055. doi: 10.3390/nu16132055. 38999802 PMC11243469

[33] Melali H, Abdolahi A, Sheikhbahaei E, et al. Impact of probiotics on gastrointestinal function and metabolic status after roux-en-Y gastric bypass: a double-blind, randomized trial. Obes Surg. 2024;34(6):2033–2041. doi: 10.1007/s11695-024-07225-1. 38653887

[34] Karbaschian Z, Mokhtari Z, Pazouki A, et al. Probiotic supplementation in morbid obese patients undergoing one anastomosis gastric bypass-mini gastric bypass (OAGB-MGB) surgery: a randomized, double-blind, placebo-controlled, clinical trial. Obes Surg. 2018;28(9):2874–2885. doi: 10.1007/s11695-018-3280-2. 29725975

[35] Ghafouri-Taleghani F, Tafreshi AS, Doost AH, et al. Effects of probiotic supplementation added to a weight loss program on anthropometric measures, body composition, eating behavior, and related hormone levels in patients with food addiction and weight regain after bariatric surgery: a randomized clinical trial. Obes Surg. 2024;34(9):3181–3194. doi: 10.1007/s11695-024-07437-5. 39117856

[36] Fernandes R, Beserra BT, Mocellin MC, et al. Effects of prebiotic and synbiotic supplementation on inflammatory markers and anthropometric indices after roux-en-Y gastric bypass: a randomized, triple-blind, placebo-controlled pilot study. J Clin Gastroenterol. 2016;50(3):208–217. doi: 10.1097/MCG.0000000000000328. 25909598

[37] Dowgiałło-Gornowicz N, Mysiorska D, Sosnowska-Turek E, et al. Initial study on the impact of probiotics on postoperative gastrointestinal symptoms and gut microbiota after sleeve gastrectomy: a placebo-controlled study. Nutrients. 2024;16(20):3498. doi: 10.3390/nu16203498. 39458493 PMC11510060

[38] Crommen S, Rheinwalt KP, Plamper A, et al. A specifically tailored multistrain probiotic and micronutrient mixture affects nonalcoholic fatty liver disease—related markers in patients with obesity after mini gastric bypass surgery. J Nutr. 2022;152(2):408–418. doi: 10.1093/jn/nxab392. 34919684

[39] Carlos L, Ramos MRZ, Wagner NRF, et al. Probiotic supplementation attenuates binge eating and food addiction 1 year after roux-En-Y gastric bypass: a randomized, double-blind, placebo-controlled trial. ABCD, Arq Bras Cir Dig. 2022;35 doi: 10.1590/0102-672020210002e1659. PMC9254603 35766604

[40] Woodard GA, Encarnacion B, Downey JR, et al. Probiotics improve outcomes after roux-en-Y gastric bypass surgery: a prospective randomized trial. J Gastrointest Surg. 2009;13(7):1198–1204. doi: 10.1007/s11605-009-0891-x. 19381735

[41] Zhang Y, Yan T, Xu C, et al. Probiotics can further reduce waist circumference in adults with morbid obesity after bariatric surgery: a systematic review and meta-analysis of randomized controlled trials. Evid Based Compl Alternat Med. 2021;2021:5542626. doi: 10.1155/2021/5542626. PMC8032506 33859706

[42] Chen IW, Hung K-C. Impact of probiotics on triglyceride level after bariatric surgery: a trial sequential analysis. Obes Surg. 2025;35(2):651–654. doi: 10.1007/s11695-025-07670-6. 39794662

[43] Mukherjee S, Joardar N, Sengupta S, et al. Gut microbes as future therapeutics in treating inflammatory and infectious diseases: lessons from recent findings. J Nutr Biochem. 2018;61:111–128. doi: 10.1016/j.jnutbio.2018.07.010. 30196243 PMC7126101

[44] Maestri M, Santopaolo F, Pompili M, et al. Gut microbiota modulation in patients with non-alcoholic fatty liver disease: effects of current treatments and future strategies. Front Nutr. 2023;10:1110536. doi: 10.3389/fnut.2023.1110536. 36875849 PMC9978194

[45] Martinez KB, Pierre JF, Chang EB. The gut microbiota: the gateway to improved metabolism. Gastroenterol Clin North Am. 2016;45(4):601–614. doi: 10.1016/j.gtc.2016.07.001. 27837775 PMC5127273

[46] Salazar J, Angarita L, Morillo V, et al. Microbiota and diabetes mellitus: role of lipid mediators. Nutrients. 2020;12(10):3039. doi: 10.3390/nu12103039. 33023000 PMC7600362

[47] Chen C, Gao K, Chen Z, et al. The supplementation of the multi-strain probiotics WHHPRO^™^ alleviates high-fat diet-induced metabolic symptoms in rats via gut-liver axis. Front Nutr. 2023;10:1324691. doi: 10.3389/fnut.2023.1324691. 38274203 PMC10808617

[48] Herbella FA, Vicentine FP, Del Grande JC, et al. Postprandial proximal gastric acid pocket in patients after Roux-en-Y gastric bypass. J Gastrointest Surg. 2010;14(11):1742–1745. doi: 10.1007/s11605-010-1309-5. 20717738

[49] Gumbs AA, Gagner M, Dakin G, et al. Sleeve gastrectomy for morbid obesity. Obes Surg. 2007;17(7):962–969. doi: 10.1007/s11695-007-9151-x. 17894158

[50] Hamamah S, Hajnal A, Covasa M. Influence of bariatric surgery on gut microbiota composition and its implication on brain and peripheral targets. Nutrients. 2024;16(7):1071. doi: 10.3390/nu16071071. 38613104 PMC11013759

[51] Voermans B, Gerdes V, Nieuwdorp M. Gut microbiota alterations and their role in the pathophysiology of obesity following bariatric surgery. Expert Rev Endocrinol Metab. 2025;20(4):291–305. doi: 10.1080/17446651.2025.2512551. 40460250

[52] Chen JC, Lee WJ, Tsou JJ, et al. Effect of probiotics on postoperative quality of gastric bypass surgeries: a prospective randomized trial. Surg Obes Relat Dis. 2016;12(1):57–61. doi: 10.1016/j.soard.2015.07.010. 26499352

[53] Han ML, Lee MH, Lee WJ, et al. Probiotics for gallstone prevention in patients with bariatric surgery: A prospective randomized trial. Asian J Surg. 2022;45(12):2664–2669. doi: 10.1016/j.asjsur.2022.01.120. 35232647

[54] Wagner NRF, Fernandes R, Teixeira Frota Reichmann M, et al. Use of probiotics and synbiotics in the treatment of small intestinal bacterial overgrowth (SIBO) and other gastrointestinal symptoms after metabolic bariatric surgery: a systematic review and meta-analysis. Obes Surg. 2025;35(1):312–321. doi: 10.1007/s11695-024-07599-2. 39607556

[55] Nuzzo A, Czernichow S, Hertig A, et al. Prevention and treatment of nutritional complications after bariatric surgery. Lancet Gastroenterol Hepatol. 2021;6(3):238–251. doi: 10.1016/S2468-1253(20)30331-9. 33581762

[56] Huang YP, Shi JY, Luo XT, et al. How do probiotics alleviate constipation? A narrative review of mechanisms. Crit Rev Biotechnol. 2025;45(1):80–96. doi: 10.1080/07388551.2024.2336531. 38710624

[57] Smolinska S, Popescu FD, Zemelka-Wiacek M. A review of the influence of prebiotics, probiotics, synbiotics, and postbiotics on the human gut microbiome and intestinal integrity. J Clin Med. 2025;14(11):3673. doi: 10.3390/jcm14113673. 40507435 PMC12156228

[58] Xiang Q, Yu M, Cai Q, et al. Multi-omics insights into the microbiota-gut-brain axis and cognitive improvement post-bariatric surgery. J Transl Med. 2024;22(1):945. doi: 10.1186/s12967-024-05757-9. 39420319 PMC11484437

[59] Münzker J, Haase N, Till A, et al. Functional changes of the gastric bypass microbiota reactivate thermogenic adipose tissue and systemic glucose control via intestinal FXR-TGR5 crosstalk in diet-induced obesity. Microbiome. 2022;10(1):96. doi: 10.1186/s40168-022-01264-5. 35739571 PMC9229785

