# Supplementary figures and images for: The effect of probiotics on weight management in patients with severe obesity undergoing metabolic and bariatric surgery: a systematic review and meta-analysis
Source: Ann Med. 2025 Aug 24;57(1):2551284. doi: 10.1080/07853890.2025.2551284 (PMC12379696; doi:10.1080/07853890.2025.2551284)

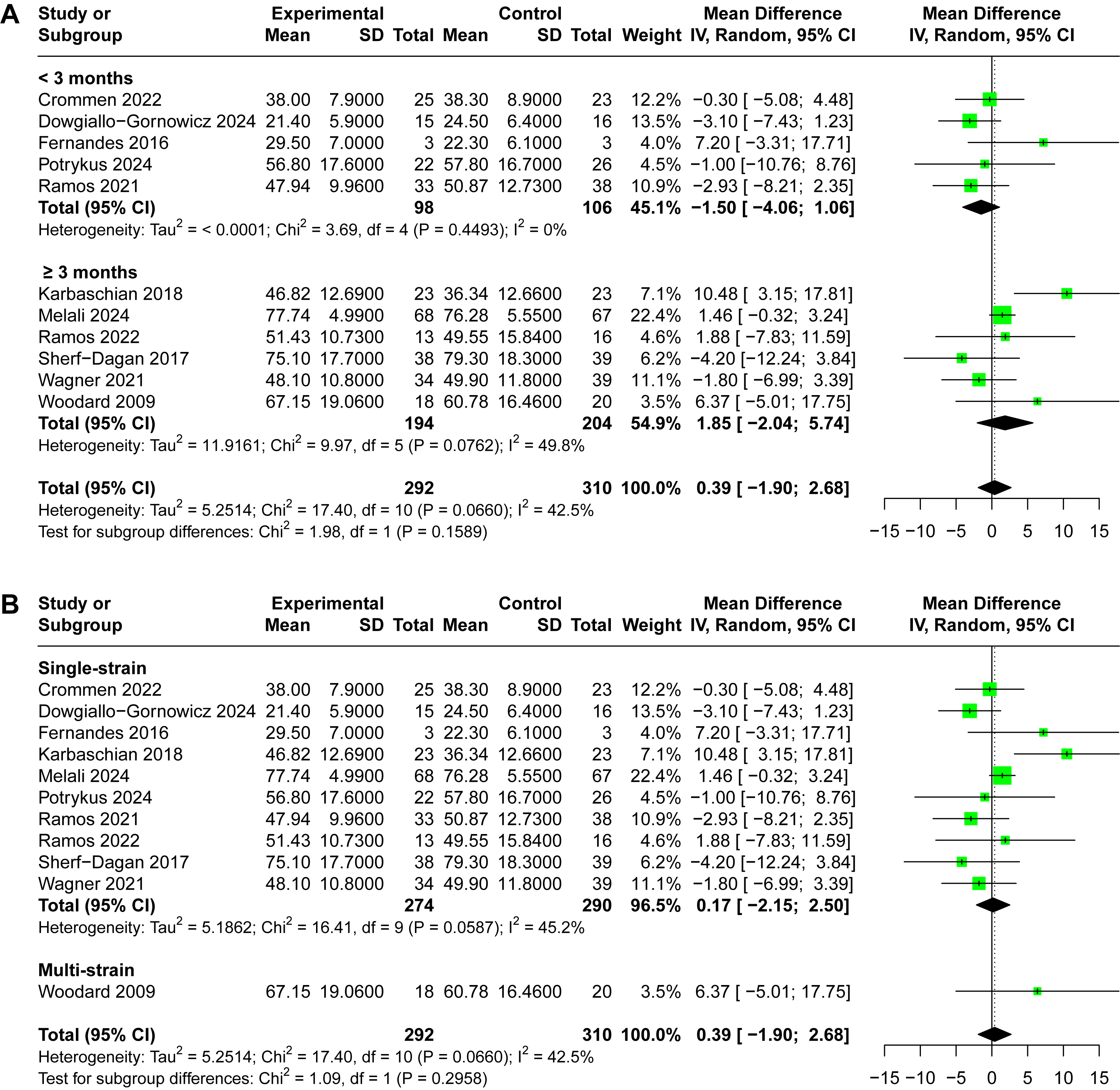

Supplement: sup5subewl.tif [file IANN_A_2551284_SM5707.tif]

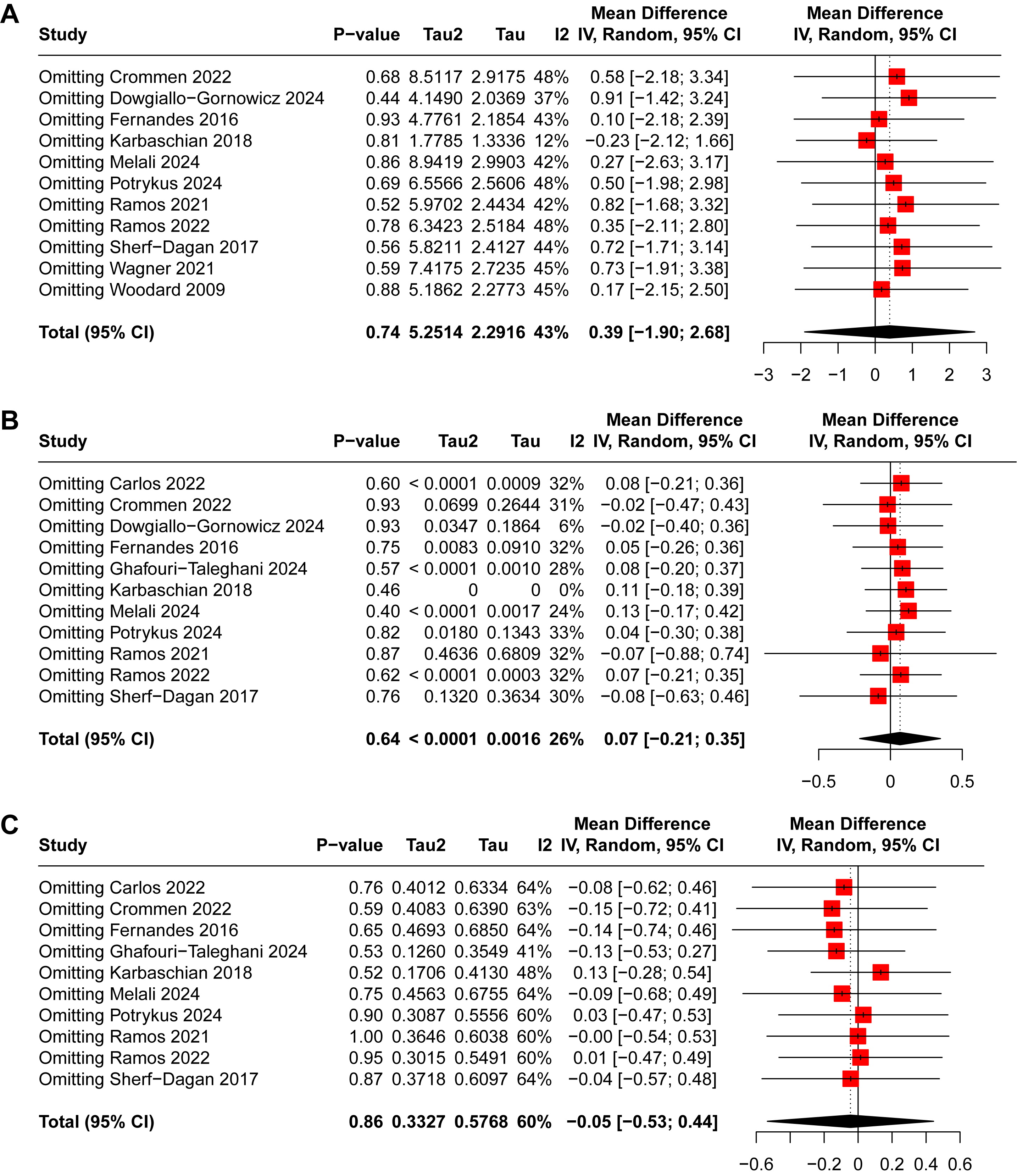

Supplement: sup4 sen.tif [file IANN_A_2551284_SM5705.tif]

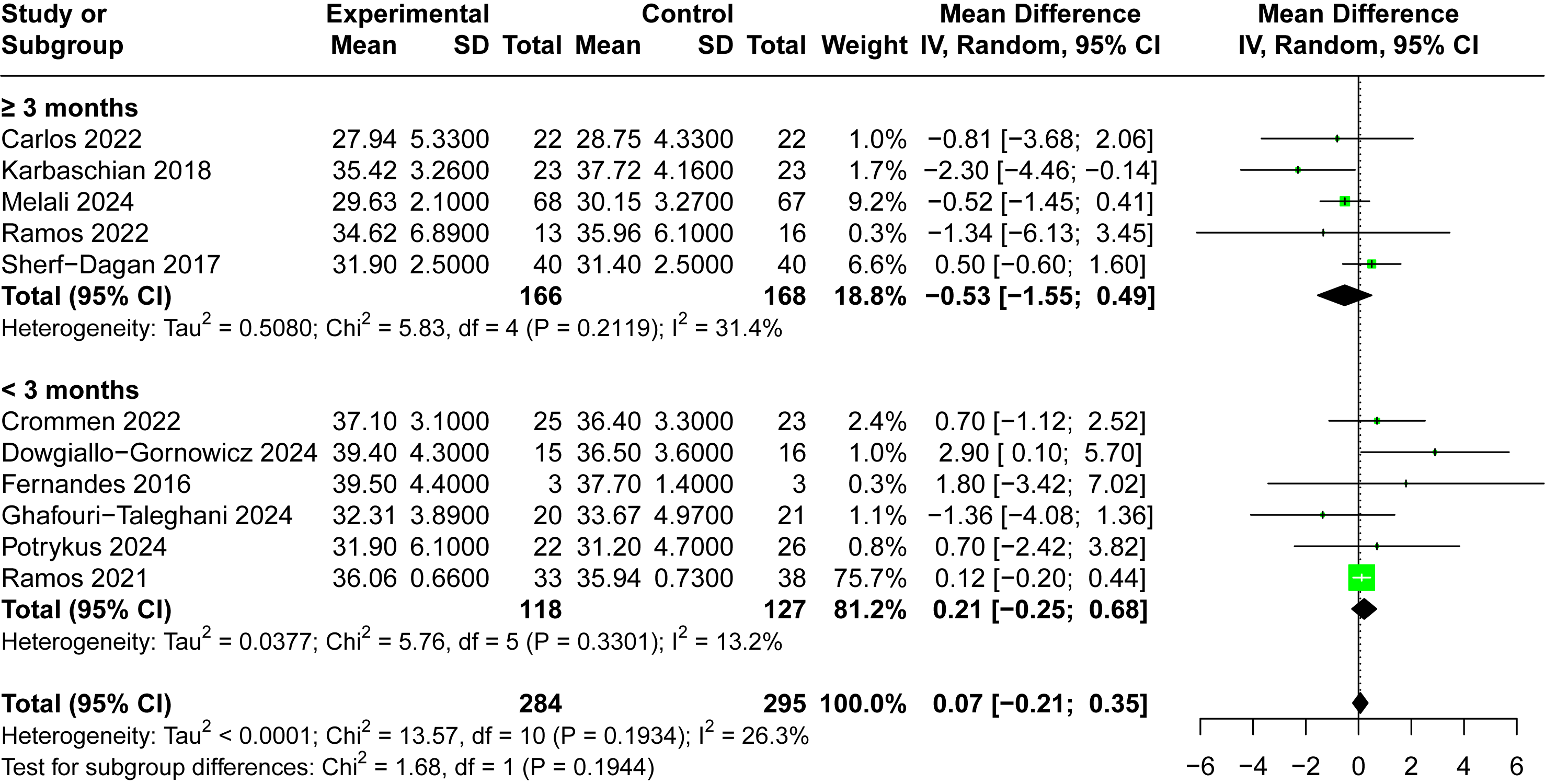

Supplement: sup6subbmi.tif [file IANN_A_2551284_SM5703.tif]

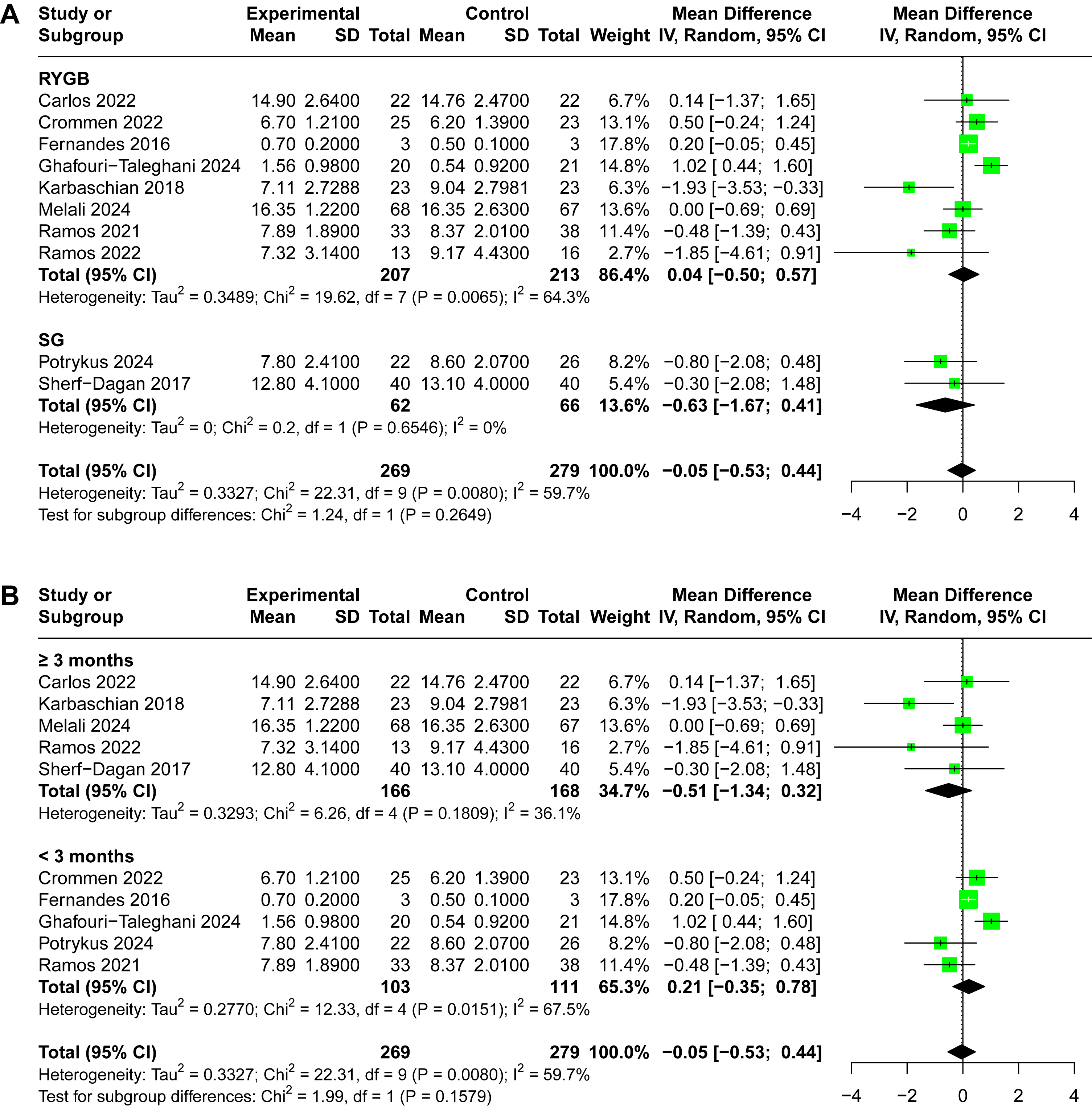

Supplement: sup7subbmi2.tif [file IANN_A_2551284_SM5702.tif]

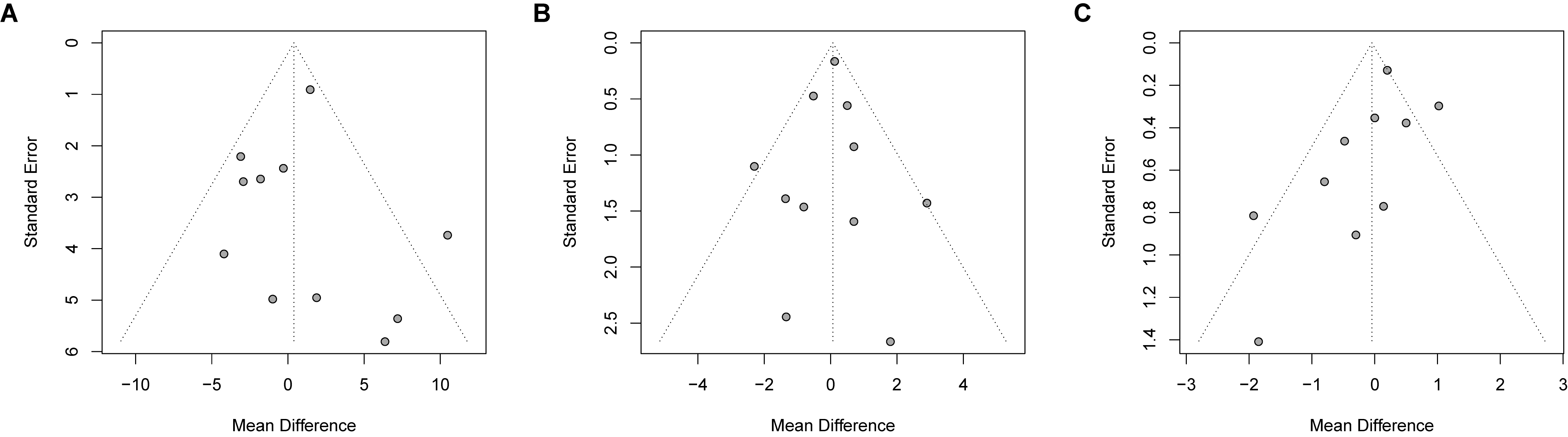

Supplement: sup3 funnel.tif [file IANN_A_2551284_SM5701.tif]
