# Supplementary material for: The effect of probiotics on weight management in patients with severe obesity undergoing metabolic and bariatric surgery: a systematic review and meta-analysis
Source: Ann Med. 2025 Aug 24;57(1):2551284. doi: 10.1080/07853890.2025.2551284 (PMC12379696; doi:10.1080/07853890.2025.2551284)
Supplement: sfile2 search.docx [file IANN_A_2551284_SM5706.docx]

**Supplementary Material 2:** Searching strategies

**Pubmed**

#1 probiotic* [Title/Abstract] OR prebiotic* [Title/Abstract] OR symbiotic [Title/Abstract] OR synbiotics [Title/Abstract] OR Probiotics [MeSH]

#2 gastric bypass [Title/Abstract] OR gastric bypass [MeSH] OR bariatric [Title/Abstract] OR gastrectomy [MeSH] OR gastrectomy [Title/Abstract]

#3 random*[Title/Abstract] OR randomized control trial [Publication Type]

#1 AND #2 AND #3

**Embase**

#1 ‘probiotic*’:ab,ti OR ‘prebiotic*’:ab,ti OR ‘symbiotic’:ab,ti OR ‘synbiotics’:ab,ti

#2 ‘gastric bypass’:ab,ti OR ‘bariatric’:ab,ti OR ‘gastrectomy’:ab,ti

#3 ‘randomized control trial’:ab,ti OR ‘random*’:ab,ti

#1 AND #2 AND #3

**Scopus**

#1 TITLE-ABS-KEY (probiotic*) OR TITLE-ABS-KEY (prebiotic*) OR TITLE-ABS-KEY (symbiotic) OR TITLE-ABS-KEY (synbiotics)

#2 TITLE-ABS-KEY (gastric bypass) OR TITLE-ABS-KEY (bariatric) OR TITLE-ABS-KEY (gastrectomy)

#3 TITLE-ABS-KEY (random*) OR TITLE-ABS-KEY (randomized control trial)

#1 AND #2 AND #3

**Cochrane Library**

#1 ‘probiotic*’:ti,ab,kw OR ‘prebiotic*’:ti,ab,kw OR ‘symbiotic’:ti,ab,kw OR ‘synbiotics’:ti,ab,kw

#2 ‘gastric bypass’:ti,ab,kw OR ‘bariatric’:ti,ab,kw OR ‘gastrectomy’:ti,ab,kw

#3 ‘random*’:ti,ab,kw OR (randomized control trial):ti,ab,kw

#1 AND #2 AND #3
